# Case Report: New-Onset Rheumatoid Arthritis Following COVID-19 Vaccination

**DOI:** 10.3389/fimmu.2022.859926

**Published:** 2022-05-27

**Authors:** Tomohiro Watanabe, Kosuke Minaga, Akane Hara, Tomoe Yoshikawa, Ken Kamata, Masatoshi Kudo

**Affiliations:** Department of Gastroenterology and Hepatology, Kindai University Faculty of Medicine, Osaka-Sayama, Japan

**Keywords:** rheumatoid arthritis, COVID-19 vaccination, type I IFN, IL-6, TNF-alpha

## Abstract

Efficient protection against coronavirus disease 2019 (COVID-19) has been achieved by immunization with mRNA-based vaccines against severe acute respiratory syndrome coronavirus 2 (SARS-CoV-2). However, efficient immune responses against this novel virus by vaccination are accompanied by a wide variety of side effects. Indeed, flares or new-onset of autoimmune disorders have been reported soon after the COVID-19 vaccination. Although pro-inflammatory cytokine responses play pathogenic roles in the development of autoimmunity, cytokines charactering COVID-19 vaccination-related autoimmune responses have been poorly understood. Given that mRNA derived from COVID-19 vaccine is a potent inducer for pro-inflammatory cytokine responses, these cytokines might mediate autoimmune responses after COVID-19 vaccination. Here we report a case with new-onset rheumatoid arthritis (RA) following COVID-19 vaccination. Serum concentrations not only of arthrogenic cytokines, interleukin-6 (IL-6) and tumor necrosis factor-α (TNF-α), but also of type I interferon (IFN) were elevated at the active phase in this case. Induction of remission by methotrexate and tocilizumab was accompanied by a marked reduction in serum concentrations of type I IFN, IL-6, and TNF-α. These results suggest that production of type I IFN, IL-6, and TNF-α induced by COVID-19 vaccination might be involved in this case with new-onset RA.

## Introduction

Coronavirus disease 2019 (COVID-19) sometimes causes autoimmunity (1). Molecular mimicry has been considered to be involved in the development of autoimmunity due to cross-reactivity of a COVID-19 spike protein to human antigens (1). Given that BNT162b2 (BioNTech-Pfizer) is a mRNA-based vaccine expressing the spike protein, it is possible that BNT162b2 vaccination can be a trigger for the development of autoimmunity (2–4). In fact, new-onset or flares of autoimmune disorders including rheumatoid arthritis (RA), adult-onset Still’s disease, inflammatory bowel diseases, and anti-neutrophil cytoplasmic antibody-associated vasculitis have been observed soon after the COVID-19 vaccination (5–9). Immunopathogenesis underlying COVID-19 vaccination-related autoimmunity has not been fully understood. In this regard, sensing of double-stranded and single-stranded RNA derived from the mRNA-based vaccine by toll-like receptors (TLRs), retinoic acid inducible gene-I (RIG-I), and melanoma differentiation-associated gene 5 (MDA5) can be potent triggers for production of type I interferons (IFN-I) and pro-inflammatory mediators (2–4). Therefore, it is likely that excessive production of IFN-I and pro-inflammatory cytokines is involved in the development of autoimmune responses associated with COVID-19 vaccination. However, cytokines causing autoimmunity have not been identified in such cases. Here, we report a case with new-onset RA following COVID-19 vaccination. In this case, serum and synovial fluid concentrations of interleukin (IL)-6, tumor necrosis factor-α (TNF-α), and IFN-I were elevated, suggesting that pro-inflammatory cytokine responses triggered by COVID-19 vaccination might be involved in the development of *de novo* RA.

## Case Report

**Figure 1:**
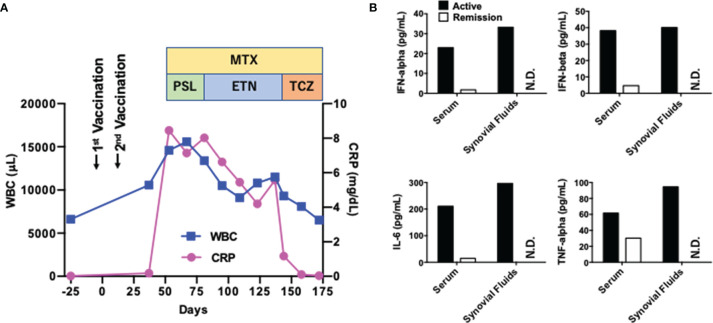
Clinical course of a patient with rheumatoid arthritis exhibiting systemic pro-inflammatory cytokine responses. **(A)** Clinical course. The first vaccination day was defined as Day 0. WBC, white blood cell; CRP, C-reactive protein; MTX, methotrexate; PSL, prednisolone; ETN, etanercept; TCZ, tocilizumab. **(B)** Serum concentrations of cytokines at the active (Day 53) and remitted (Day 172) phases. Serum concentrations of IL-6, TNF-α, IFN-α, and IFN-β at the active and remitted phases are shown. Synovial fluids were obtained at the active phase alone. N.D., not done.

A 53-year-old healthy Japanese man received BNT162b2 vaccination twice. His grandmother had RA. Successful immunization was verified by a marked elevation of anti-COVID-19-specific Ab titer (510 U/mL, normal range <15). Four weeks after the final vaccination, his left knee joint became swollen and painful. Soon after, he noticed bilateral omalgia and morning stiffness. Blood examination revealed marked leukocytosis (white blood cell, WBC count; 14,600/μL, normal range; 4,000~9,000) and elevated levels of C-reactive protein (CRP 8.45 mg/dL, normal range; 0~0.60). WBC counts and CRP levels were normal before the second vaccination (**Figure 1A**). His serum levels of anti-cyclic citrullinated peptide antibody and rheumatoid factor were 1,200 U/mL (normal range; 0~4.4) and 51 U/mL (normal range; 0~15), respectively. Magnetic resonance imaging showed diffuse knee effusion. Based on these typical symptoms and serological analyses, he was diagnosed as RA.

Initial treatment with methotrexate (MTX, 3 mg/day) with an escalating dose schedule (1 mg/2 weeks) and prednisolone (5 mg/day) failed to induce remission. PSL was switch to subcutaneous injection of etanercept (50 mg/week), which was again unsuccessful for the induction of remission. MTX (6 mg/day) in combination with subcutaneous injection of tocilizumab (162 mg/week) normalized serum concentration of CRP and WBC count (**Figure 1A**). Around one month after the injection of tocilizumab, induction of remission was achieved and bilateral omalgia, swollen of the left knee, and morning stiffness disappeared at this time point. He was treated with MTX and tocilizumab (162 mg/every two weeks) and maintained complete remission for more than ten months without adverse events.

## Methods

Concentrations of IFN-α, IFN-β, IL-6, and TNF-α were measured using enzyme-linked immunosorbent assay kits from R&D systems. Serum samples were obtained at the active and remitted phases whereas joint fluid samples were taken at the active phase.

## Results

One major question arising from this case is whether BNT162b2 vaccination promoted the development of RA. Pro-inflammatory cytokines such as IL-6 and TNF-α underlie the immuno-pathogenesis of RA whereas the IFN-I responses are not so prominent in RA (10). mRNA derived from BNT162b2 can cause strong IFN-I responses through sensing by TLRs, RIG-I, and MDA5 (2–4). Therefore, it is possible that not only IL-6 and TNF-α but also IFN-I is involved in the development of RA in this case. As expected, serum concentrations of TNF-α and IL-6 were markedly reduced after treatment with MTX and tocilizumab (**Figure 1B**). Interestingly, serum concentrations of IFN-α and IFN-β were also high before the treatment and declined at the remission phase (**Figure 1B**). In addition, synovial fluid obtained from the left knee before the treatment contained high levels of IFN-I as well as IL-6. Thus, this new-onset RA after BNT162b2 vaccination was characterized by IFN-I as well as IL-6 and TNF-α.

## Discussion

Although mRNA-based COVID-19 vaccines are very effective for the protection against SARS-CoV-2 infection, the induction of efficient immune responses is concomitant with a wide variety of side effects (2–4). New-onset or exacerbation of autoimmune disorders has been reported in patients soon after COVID-19 vaccination (5–9). Given that pro-inflammatory cytokine responses play crucial roles in the development of autoimmune disorders, it is likely that COVID-19 vaccination can be a trigger for production of pro-inflammatory cytokines. Different from conventional vaccines, mRNA-based COVID-19 vaccines do not contain adjuvants with potent immuno-stimulatory functions (2–4). In this regards, recognition of mRNAs derived from COVID-19 vaccine by TLRs, RIG-I, and MDA5 may induce pro-inflammatory cytokines, including IFN-I (2–4). However, molecular mechanisms accounting for the development of autoimmunity following COVID-19 vaccination have not been fully understood. Here we show a case of *de novo* RA following COVID-19 vaccination and characterized by systemic pro-inflammatory cytokine responses including IFN-I, IL-6, and TNF-α. Although new-onset or exacerbation of autoimmune disorders have been reported in patients soon after COVID-19 vaccination (5–9), little information was available regarding pro-inflammatory cytokine responses in such cases.

Although IL-6 and TNF-α are well-established arthrogenic cytokines, roles of IFN-I have been poorly defined. The IFN-I signature is less conspicuous in RA than in the other IFN-I-dependent autoimmunity such as systemic lupus erythematosus (11–13). Thus, this case was atypical in that IFN-I responses as well as prototypical pro-inflammatory cytokine responses were parallel to disease activities. In this regard, we speculate that BNT162b2 vaccination could be a trigger for RA development. This notion is supported by the fact that mRNA derived from BNT162b2 is a potent inducer of IFN-I responses through activation of TLRs, RIG-I, and MDA5 (2, 3). Consistent with high concentrations of IFN-I in the synovial fluid of this case, Lande et al. confirmed the presence of IFN-I in the synovial fluid of patients with RA (14). Taken together, this case suggests that initial IFN-I responses induced by BNT162b2 vaccination might trigger arthrogenic cytokine responses accounting for the development of typical RA *via* induction of IL-6 and TNF-α. This idea is fully supported by the fact that IFN-I can function as upstream cytokines with the ability to induce production of prototypical inflammatory cytokines such as IL-6 and TNF-α (15). However, we cannot exclude the possibility that the timing of RA development with regard to vaccination was coincidental. Moreover, this patient was successfully treated by the blockade of IL-6, which suggests predominant roles of this cytokine. Future studies performing large-scale epidemiological analyses in new-onset or flare RA patients following COVID-19 vaccination are required to establish its link. It would be intriguing to examine whether IFN-I as well as arthrogenic cytokine (IL-6 and TNF-α) responses are elevated in COVID-19 vaccination-associated RA.

## Data Availability Statement

The original contributions presented in the study are included in the article/supplementary material. Further inquiries can be directed to the corresponding author.

## Ethics Statement

The studies involving human participants were reviewed and approved by Kindai University Faculty of Medicine. The patients/participants provided their written informed consent to participate in this study.

## Author Contributions

TW and KM wrote the manuscript draft and measured concentrations of cytokines. TW, KM, AH, TY, KK, and MK revised the manuscript. All authors contributed to the article and approved the submitted version.

## Funding

This study was supported in part by a Grant-in-Aid for Scientific Research (22K07996) from Japan Society for the Promotion of Science, Yakult Bioscience Foundation, Smoking Research Foundation and Takeda Science Foundation.

## Conflict of Interest

The authors declare that the research was conducted in the absence of any commercial or financial relationships that could be construed as a potential conflict of interest.

## Publisher’s Note

All claims expressed in this article are solely those of the authors and do not necessarily represent those of their affiliated organizations, or those of the publisher, the editors and the reviewers. Any product that may be evaluated in this article, or claim that may be made by its manufacturer, is not guaranteed or endorsed by the publisher.

## References

[1] Ehrenfeld M Tincani A Andreoli L Cattalini M Greenbaum A Kanduc D. Covid-19 and Autoimmunity. Autoimmun Rev (2020) 19(8):102597. doi: 10.1016/j.autrev.2020.102597 32535093 PMC7289100

[2] Teijaro JR Farber DL. COVID-19 Vaccines: Modes of Immune Activation and Future Challenges. Nat Rev Immunol (2021) 21(4):195–7. doi: 10.1038/s41577-021-00526-x PMC7934118 33674759

[3] Sprent J King C. COVID-19 Vaccine Side Effects: The Positives About Feeling Bad. Sci Immunol (2021) 6(60). doi: 10.1126/sciimmunol.abj9256 PMC9267253 34158390

[4] Sahin U Kariko K Tureci O. mRNA-Based Therapeutics–Developing a New Class of Drugs. Nat Rev Drug Discovery (2014) 13(10):759–80. doi: 10.1038/nrd4278 25233993

[5] Hakroush S Tampe B. Case Report: ANCA-Associated Vasculitis Presenting With Rhabdomyolysis and Pauci-Immune Crescentic Glomerulonephritis After Pfizer-BioNTech COVID-19 mRNA Vaccination. Front Immunol (2021) 12:762006. doi: 10.3389/fimmu.2021.762006 34659268 PMC8514980

[6] Leone F Cerasuolo PG Bosello SL Verardi L Fiori E Cocciolillo F. Adult-Onset Still's Disease Following COVID-19 Vaccination. Lancet Rheumatol (2021) 3(10):e678–80. doi: 10.1016/S2665-9913(21)00218-6 PMC8298028 34316728

[7] Weaver KN Zhang X Dai X Watkins R Adler J Dubinsky MC. Impact of SARS-CoV-2 Vaccination on Inflammatory Bowel Disease Activity and Development of Vaccine-Related Adverse Events: Results From PREVENT-COVID. Inflamm Bowel Dis (2021). doi: 10.1093/ibd/izab302 PMC8822409 34871388

[8] Sangha M Roitman I Sultan K Swaminath A. SARS-CoV-2 Immunization in Patients With Inflammatory Bowel Disease May Result in Disease Flares. Am J Gastroenterol (2021) 116(12):2480–1. doi: 10.14309/ajg.0000000000001416 34406140

[9] Terracina KA Tan FK. Flare of Rheumatoid Arthritis After COVID-19 Vaccination. Lancet Rheumatol (2021) 3(7):e469–70. doi: 10.1016/S2665-9913(21)00108-9 PMC8009616 33817664

[10] McInnes IB Schett G. Cytokines in the Pathogenesis of Rheumatoid Arthritis. Nat Rev Immunol (2007) 7(6):429–42. doi: 10.1038/nri2094 17525752

[11] Higgs BW Liu Z White B Zhu W White WI Morehouse C. Patients With Systemic Lupus Erythematosus, Myositis, Rheumatoid Arthritis and Scleroderma Share Activation of a Common Type I Interferon Pathway. Ann Rheum Dis (2011) 70(11):2029–36. doi: 10.1136/ard.2011.150326 21803750

[12] Watanabe T Minaga K Kamata K Kudo M Strober W. Mechanistic Insights Into Autoimmune Pancreatitis and IgG4-Related Disease. Trends Immunol (2018) 39(11):874–89. doi: 10.1016/j.it.2018.09.005 30401468

[13] Chasset F Dayer JM Chizzolini C. Type I Interferons in Systemic Autoimmune Diseases: Distinguishing Between Afferent and Efferent Functions for Precision Medicine and Individualized Treatment. Front Pharmacol (2021) 12:633821. doi: 10.3389/fphar.2021.633821 33986670 PMC8112244

[14] Lande R Giacomini E Serafini B Rosicarelli B Sebastiani GD Minisola G. Characterization and Recruitment of Plasmacytoid Dendritic Cells in Synovial Fluid and Tissue of Patients With Chronic Inflammatory Arthritis. J Immunol (2004) 173(4):2815–24. doi: 10.4049/jimmunol.173.4.2815 15295000

[15] Watanabe T Sadakane Y Yagama N Sakurai T Ezoe H Kudo M. Nucleotide-Binding Oligomerization Domain 1 Acts in Concert With the Cholecystokinin Receptor Agonist, Cerulein, to Induce IL-33-Dependent Chronic Pancreatitis. Mucosal Immunol (2016) 9(5):1234–49. doi: 10.1038/mi.2015.144 26813347

